# Supplementary material for: Parental Educational Intervention to Facilitate Informed Consent for Pediatric Procedural Sedation in the Emergency Department: A Parallel-Group Randomized Controlled Trial
Source: Healthcare (Basel). 2022 Nov 23;10(12):2353. doi: 10.3390/healthcare10122353 (PMC9778183; doi:10.3390/healthcare10122353)
Supplement: Supplementary file 1 [file healthcare-10-02353-s001.zip › Table S1.pdf]

Table S1. Basic characteristics

Characteristics	Conventional group (n=30)	Video group (n=32)	*p*-value
Age (years)	number (%)	number (%)	0.109[1]
20–29	6 (20.0)	8 (25.0)	
30–39	17 (56.7)	23 (71.9)	
40–49	6 (20.0)	1 (3.1)	
50–59	1 (3.3)	0 (0.0)	
Male	7 (23.3)	8 (25.0)	0.878
Education			0.928[1]
<High school	3 (10.0)	3 (9.4)	
High school	10 (33.3)	9 (28.1)	
College	17 (56.7)	20 (62.5)	
Arrival time, 8–16 h	12 (40.0)	9 (28.1)	0.423[1]
Physician			0.847[1]
Physician A	4 (13.3)	5 (15.6)	
Physician B	5 (16.7)	4 (12.5)	
Physician C	4 (13.3)	8 (25.0)	
Physician D	7 (23.3)	6 (18.8)	
Physician E	5 (16.7)	6 (18.7)	
Physician F	5 (16.7)	3 (9.4)	

[1] Fisher's exact test
