# Supplementary material for: Parental Educational Intervention to Facilitate Informed Consent for Pediatric Procedural Sedation in the Emergency Department: A Parallel-Group Randomized Controlled Trial
Source: Healthcare (Basel). 2022 Nov 23;10(12):2353. doi: 10.3390/healthcare10122353 (PMC9778183; doi:10.3390/healthcare10122353)
Supplement: Supplementary file 1 [file healthcare-10-02353-s001.zip › Table S2.pdf]

Table S2. Categorized knowledge score differences.

Knowledge score difference	Conventional group (n=30)	Video group (n=32)	*p*-value[2]
	Number (%)	Number (%)	
≤20	20 (66.67)	9 (28.13)	0.002[1]
21–49	8 (26.67)	10 (31.25)	
≥50	2 (6.67)	13 (40.63)	

[1] Fisher's exact test.

[2] Video versus conventional.
