# Supplementary material for: Parental Educational Intervention to Facilitate Informed Consent for Pediatric Procedural Sedation in the Emergency Department: A Parallel-Group Randomized Controlled Trial
Source: Healthcare (Basel). 2022 Nov 23;10(12):2353. doi: 10.3390/healthcare10122353 (PMC9778183; doi:10.3390/healthcare10122353)
Supplement: Supplementary file 1 [file healthcare-10-02353-s001.zip › Table S3.pdf]

Table S3. Comparison of baseline knowledge between the conventional and video subgroups.

Variable	Conventional group			Video group			*p*-value
	n	Mean	Standard deviation	n	Mean	Standard deviation	
Age (years)							
<34	16	54.17	19.72	17	53.92	17.21	0.970
≥34	14	51.19	20.11	15	55.56	16.27	0.525
Sex							
Female	23	55.07	19.74	24	54.17	17.20	0.867
Male	7	45.24	18.55	8	56.25	15.27	0.229
Education							
<College	13	37.18	13.87	12	44.44	14.80	0.218
≥College	17	64.70	14.29	20	60.83	14.59	0.422
Arrival time							
08:00–16:00 h	12	58.33	19.46	9	59.26	16.90	0.910
Others	18	49.08	19.36	23	52.90	16.40	0.498
Physician							
Physician A	4	62.50	15.96	5	56.67	9.13	0.510
Physician B	5	53.33	13.95	4	58.34	16.67	0.638
Physician C	4	45.83	15.96	8	45.83	14.78	1.000
Physician D	7	50.00	16.67	6	58.33	17.48	0.398
Physician E	5	56.67	25.27	6	63.89	19.48	0.605
Physician F	5	45.00	31.18	3	44.44	19.25	0.793
