# Supplementary material for: Parental Educational Intervention to Facilitate Informed Consent for Pediatric Procedural Sedation in the Emergency Department: A Parallel-Group Randomized Controlled Trial
Source: Healthcare (Basel). 2022 Nov 23;10(12):2353. doi: 10.3390/healthcare10122353 (PMC9778183; doi:10.3390/healthcare10122353)
Supplement: Supplementary file 1 [file healthcare-10-02353-s001.zip › Table S4.pdf]

Table S4. Comparison of post-education knowledge scores between conventional and video subgroups.

Variable	Conventional group			Video group			*p*-value
	n	Mean	Standard deviation	n	Mean	Standard deviation	
Age (years)							
<34	16	72.92	19.12	17	95.10	9.80	<0.001***
≥34	14	73.81	21.40	15	87.78	14.73	0.049*
Sex							
Female	23	74.64	20.02	24	94.44	9.41	<0.001***
Male	7	69.05	20.25	8	83.33	17.82	0.170
Education							
<College	13	64.10	19.06	12	84.72	16.60	0.009**
≥College	17	80.39	17.91	20	95.83	7.41	0.001**
Arrival time							
08:00–16:00 h	12	80.55	21.12	9	90.74	16.90	0.250
Others	18	68.52	17.98	23	92.03	11.09	<0.001***
Physician							
Physician A	4	75.00	21.52	5	90.00	22.36	0.343
Physician B	5	80.00	7.45	4	95.83	8.33	0.020*
Physician C	4	58.33	21.52	8	89.58	15.27	0.015*
Physician D	7	71.43	23.00	6	88.89	8.61	0.108
Physician E	5	83.33	11.78	6	94.44	8.61	0.104
Physician F	5	70.00	12.25	3	94.44	9.62	0.197

* $p < 0.05$; ** $p < 0.01$; *** $p < 0.001$.
